# Left ventricular outflow tract aneurysm in postoperative isolated ventricular inversion

**DOI:** 10.1186/s44348-024-00012-7

**Published:** 2024-06-17

**Authors:** Sudipta Mondal, Arun Gopalakrishnan, Ankita Singh, Jineesh Valakkada

**Affiliations:** 1 Department of Cardiology, Sree Chitra Tirunal Institute for Medical Sciences and Technology, Medical College P O, Thiruvananthapuram, Kerala 695011 India; 2 Department of Imaging Sciences & Intervention Radiology, Sree Chitra Tirunal Institute for Medical Sciences and Technology, Thiruvananthapuram, Kerala India

**Fig. 1:**
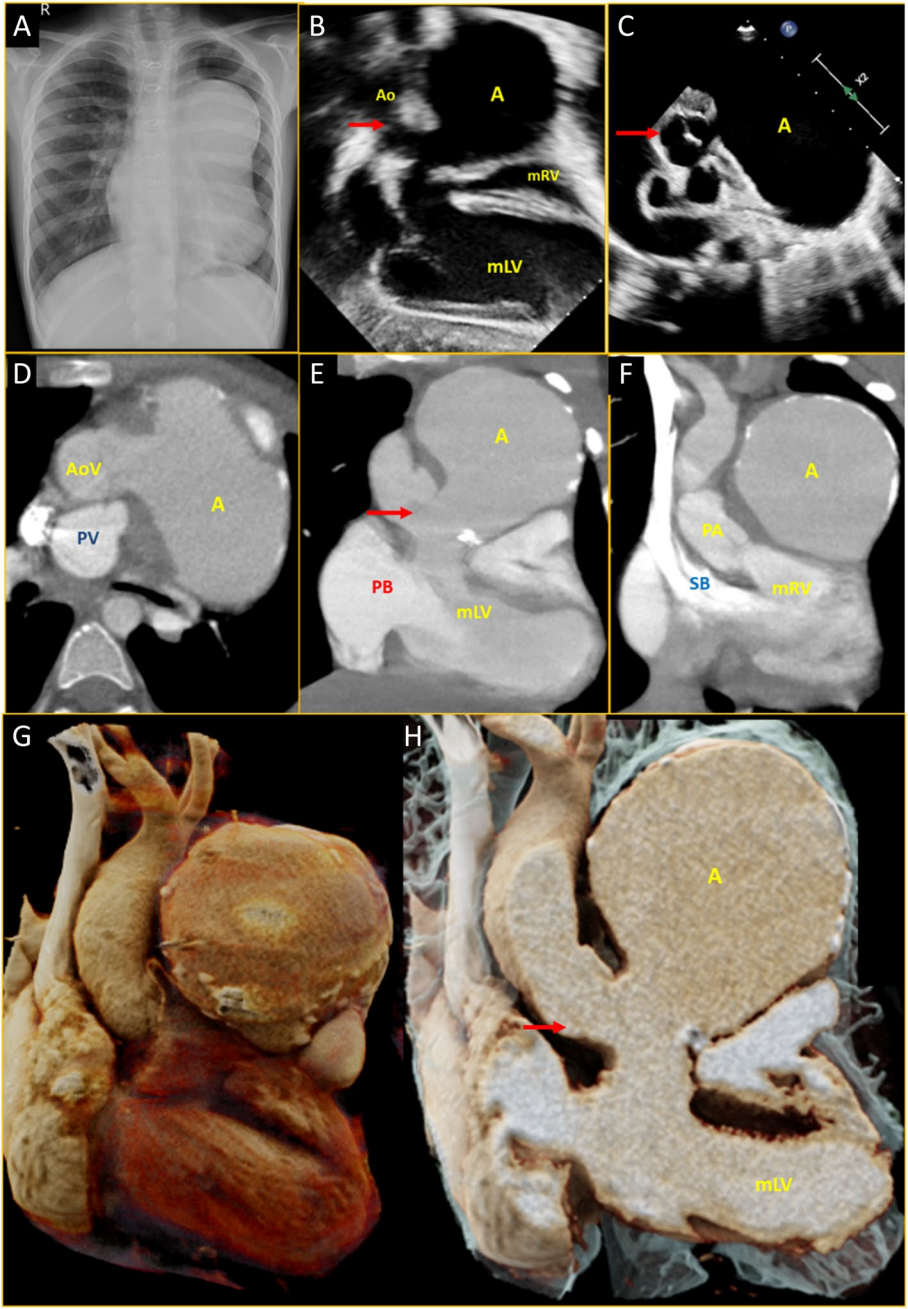
**A** Three-year post-operative chest X-ray showing huge para-cardiac mass. **B** Transthoracic echocardiogram subxiphoid view showing right sided morphological left ventricle connected to aorta with huge aneurysm just beneath the valve (red arrow). **C** Basal short axis view showing aortic valve with huge aneurysm arising from LVOT just below the aortic valve (red arrow). **D** Cardiac CT axial section showing aorta is anterior to pulmonary artery and aneurysm is sub-valvular. **E** Cardiac CT in coronal section showing right atrium with pulmonary baffle communicating with right sided morphological left ventricle, in turn connected to aorta with huge sub-valvular (aortic valve: red arrow) LVOT tunnel aneurysm. **F** Coronal section showing systemic baffle connected to left sided morphological right ventricle in turn connected to pulmonary artery. Note that: before Senning operation, left atrium was connected to left sided right ventricle, confirming ventricular inversion. **G**, **H** Volume rendered technique showing the spatial relationship of the aneurysm. A: aneurysm, Ao: aorta, AoV: aortic valve, CT: computed tomography, LVOT: left ventricular outflow tract, mLV: morphological left ventricular, mRV: morphological right ventricular, PB: pulmonary baffle, PV: pulmonary valve, SB: systemic baffle

A boy with isolated ventricular inversion, large sub-aortic ventricular septal defect (VSD) and severe infundibular pulmonary stenosis underwent a Senning procedure, VSD closure, intracardiac tunnelling of left ventricle to aorta and right ventricular outflow tract patch repair at 3 years of life. He remained asymptomatic thereafter. Clinical examination was normal and X-ray chest was unremarkable at 1-year follow-up. He missed his subsequent review and presented at 6 years of life (3rd postoperative year) for review. A left precordial bulge was noted, and a chest X-ray revealed a prominent left para-cardiac shadow (Fig. 1A). The echocardiogram showed a huge cystic mass just below the aortic valve arising from the left ventricular outflow tract (LVOT) (Fig. 1B and C, Movies 1 and 2). Cardiac CT showed no baffle leak, no residual VSD or outflow obstruction. An 8 cm × 8 cm partially calcified true aneurysm arising from the lateral wall of the LVOT was noted just beneath the aortic valve abutting the sternum (Fig. 1D-H, Movies 3, 4 and 5). The patient underwent surgical repair of the aneurysm by a 2-patch technique. The mitral and aortic valves and right ventricular outflow tract patch were noted to be normal, with no features of endocarditis. The patient remained well at the 6-month review, and the follow-up chest X-ray was normal.

LVOT aneurysm is a rare but potentially life-threatening complication of surgical trauma or endocarditis [1]. It is usually located in the mitral-aortic intervalvular fibrosa (MAIVF). The MAIVF is a relatively avascular membranous and thinned fragile structure compared to the adjacent cardiac walls and is prone to the development of aneurysm formation. Microscopic studies have also proved that the mitral fibrous body forms an incomplete ring and is deficient in collagenous material in its anteromedial aspect [2]. This is the largest documented aneurysm of MAIVF in literature and the first instance in isolated ventricular inversion [3, 4]. This case reinforces the need for continued long-term follow-up of patients post-cardiac surgery, even if asymptomatic. Chest roentgenogram remains a simple and relevant investigation for follow-up of patients after complex cardiac surgery.

### Supplementary Information

**Additional file 1: Movie 1.** Transthoracic echocardiogram subxiphoid view.

**Additional file 2: Movie 2.** Transthoracic echocardiogram basal short axis view.

**Additional file 3: Movie 3.** Cardiac CT axial section.

**Additional file 4: Movie 4.** Cardiac CT in coronal section.

**Additional file 5: Movie 5.** Cardiac CT in sagittal section.
